# Development of a GIN11/FRT-based multiple-gene integration technique affording inhibitor-tolerant, hemicellulolytic, xylose-utilizing abilities to industrial *Saccharomyces cerevisiae* strains for ethanol production from undetoxified lignocellulosic hemicelluloses

**DOI:** 10.1186/s12934-014-0145-9

**Published:** 2014-10-12

**Authors:** Tomohisa Hasunuma, Yoshimi Hori, Takatoshi Sakamoto, Misa Ochiai, Haruyo Hatanaka, Akihiko Kondo

**Affiliations:** Organization of Advanced Science and Technology, Kobe University, 1-1 Rokkodai, Nada, Kobe, 657-8501 Japan; Department of Chemical Science and Engineering, Graduate School of Engineering, Kobe University, 1-1 Rokkodai, Nada, Kobe, 657-8501 Japan; Suntory Research Center, 1-1-1 Wakayamadai, Shimamoto-cho, Osaka, Mishima-gun 618-8503 Japan; Biomass Engineering Program, RIKEN, 1-7-22 Suehiro-cho, Tsurumi-ku, Yokohama, Kanagawa 230-0045 Japan

**Keywords:** Bioethanol, Cell surface display, Inhibitor tolerance, Lignocellulose, Maker recycling, *Saccharomyces cerevisiae*

## Abstract

**Background:**

Bioethanol produced by the yeast *Saccharomyces cerevisiae* is currently one of the most promising alternatives to conventional transport fuels. Lignocellulosic hemicelluloses obtained after hydrothermal pretreatment are important feedstock for bioethanol production. However, hemicellulosic materials cannot be directly fermented by yeast: xylan backbone of hemicelluloses must first be hydrolyzed by heterologous hemicellulases to release xylose, and the yeast must then ferment xylose in the presence of fermentation inhibitors generated during the pretreatment.

**Results:**

A GIN11/FRT-based multiple-gene integration system was developed for introducing multiple functions into the recombinant *S. cerevisiae* strains engineered with the xylose metabolic pathway. Antibiotic markers were efficiently recycled by a novel counter selection strategy using galactose-induced expression of both FLP recombinase gene and *GIN11* flanked by FLP recombinase recognition target (FRT) sequences. Nine genes were functionally expressed in an industrial diploid strain of *S. cerevisiae*: endoxylanase gene from *Trichoderma reesei*, xylosidase gene from *Aspergillus oryzae*, β-glucosidase gene from *Aspergillus aculeatus*, xylose reductase and xylitol dehydrogenase genes from *Scheffersomyces stipitis*, and *XKS1*, *TAL1*, *FDH1* and *ADH1* variant from *S. cerevisiae.* The genes were introduced using the homozygous integration system and afforded hemicellulolytic, xylose-assimilating and inhibitor-tolerant abilities to the strain. The engineered yeast strain demonstrated 2.7-fold higher ethanol titer from hemicellulosic material than a xylose-assimilating yeast strain. Furthermore, hemicellulolytic enzymes displayed on the yeast cell surface hydrolyzed hemicelluloses that were not hydrolyzed by a commercial enzyme, leading to increased sugar utilization for improved ethanol production.

**Conclusions:**

The multifunctional yeast strain, developed using a GIN11/FRT-based marker recycling system, achieved direct conversion of hemicellulosic biomass to ethanol without the addition of exogenous hemicellulolytic enzymes. No detoxification processes were required. The multiple-gene integration technique is a powerful approach for introducing and improving the biomass fermentation ability of industrial diploid *S. cerevisiae* strains.

**Electronic supplementary material:**

The online version of this article (doi:10.1186/s12934-014-0145-9) contains supplementary material, which is available to authorized users.

## Background

Environmental concerns and shrinking oil reserves have resulted in governmental incentives to develop environmentally benign and sustainable fuels. The utilization of lignocellulosic biomass for the production of fuels has received particular attention in recent years. Bioethanol is produced by the fermentation of biomass by the yeast *Saccharomyces cerevisiae* and is currently one of the most promising alternatives to conventional transport fuels. *S. cerevisiae* is a superior ethanol producer with demonstrated fast sugar consumption, high ethanol yield from glucose, and high resistance to ethanol. Lignocellulosic biomass such as corn stover, rice and wheat straw, sugarcane bagasse, wood chips and other agricultural residues comprise mainly cellulose, hemicelluloses and lignin. Although the composition varies with these feedstock, hemicelluloses are the second most abundant constituent of lignocellulosic biomass. If the economic success of lignocellulosic ethanol is to be realized, both cellulose and hemicelluloses must be utilized for ethanol production.

Hemicelluloses are heterologous polymers encompassing heteroxylans, xyloglucan, heteromannanns, and the mixed-linkage glucan [1]. Heteroxylans, the most relevant hemicelluloses in agriculture wastes, consist of a xylan backbone of β-1,4-linked xylose partially substituted with acetyl, glucuronosyl and arabinosyl side chains [1]. Xylan is hydrolysed to xylooligosaccharides by endoxylanase, then xylosidase hydrolyzes xylooligosaccharides to release xylose. Several bacterial and fungal species can utilize xylan as a carbon source [2], but *S. cerevisiae* cannot. Thus, many researchers have attempted to produce xylanolytic enzymes in *S. cerevisiae* strains [3,4]. Furthermore, there has been considerable effort to engineer xylose assimilation pathways in *S. cerevisiae* [5,6]. A key aspect of metabolic engineering in yeast has been the heterologous expression of genes for the initial steps of xylose assimilation catalyzed by xylose reductase (XR) and xylitol dehydrogenase (XDH) derived from *Scheffersomyces stipitis*, combined with overexpression of *S. cerevisiae* xylulokinase (XK). Therefore, the direct conversion of hemicelluloses to ethanol by *S. cerevisiae* requires that genes for both xylanolytic enzymes and xylose-assimilating enzymes must be simultaneously expressed in recombinant yeast strains.

Yeast ethanolic fermentation from lignocellulosic materials requires the utilization of sugars in the presence of toxic compounds such as acetate, formate, furfural and 5-hydroxymethylfurfural (5-HMF) released during the biomass pretreatment process [7]. In particular, xylose utilization by recombinant *S. cerevisiae* strains was severely affected by the presence of these fermentation inhibitors [8]. Several metabolic engineering approaches have been investigated to overcome this inhibitory effect and improve the fermentation capability of yeast strains in the presence of toxic compounds. A metabolomic approach identified *TAL1*, which encodes transaldolase, as being involved in ethanol production from xylose in the presence of acetate and formate [8]. Global gene expression analysis demonstrated that formate dehydrogenase gene, *FDH1*, in a XR/XDH/XK-based recombinant *S. cerevisiae* strain, was up-regulated in response to increasing formate concentrations [9]. Coexpression of *TAL1* and *FDH1* in a recombinant xylose-fermenting *S. cerevisiae* strain improved ethanol production from xylose in the presence of both acetate and formate [10], while overexpression of variant *ADH1* improves ethanol fermentation in the presence of both furfural and 5-HMF [11].

Thus, utilization of lignocellulosic hemicelluloses for ethanol production requires both the ability to ferment hemicelluloses and to tolerate toxic compounds in the hydrolysate. This requires the integration of a large number of heterologous genes into *S. cerevisiae*. Several auxotrophic and drug-resistance markers are typically used in the integration of heterologous genes to engineer *S. cerevisiae* strains, but only a limited number of marker genes are available. Industrial strains isolated under various environmental conditions such as high sugar and ethanol concentration, nitrogen starvation, acidic pH, osmotic pressure, anaerobiosis, and high temperature [12,13] are favorable for catalyzing bioethanol production, but they are not auxotrophic strains. On the other hand, the application of drug-resistance markers is not economically feasible on an industrial scale. Moreover, industrial *S. cerevisiae* strains are usually sterile polyploids, making it difficult to construct stable homozygous recombinant strains by classical genetic procedures.

We here describe the development of a GIN11/FRT-based novel marker recycling system. The *GIN11* sequence has a growth-inhibitory effect on *S. cerevisiae* when overexpressed [14]. FLP recombinase target (FRT) sequences can be targeted by FLP recombinase for pop-out genome recombination [15]. In this study, efficient counter selection based on galactose-induced expression of *GIN11* flanked by FRT sequences achieved multiple gene integrations into inter open reading frame (inter-ORF) regions in the genome of an industrial diploid *S. cerevisiae* strain. Using this system, an industrial brewer’s yeast strain was engineered to produce ethanol directly from lignocellulosic hemicelluloses prepared hydrothermally from rice straw containing fermentation inhibitors. The integration of genes for hemicellulolytic enzymes and xylose-assimilating enzymes, as well as inhibitor resistance genes, significantly improves ethanol production from biomass substrate.

## Results

### Construction of recombinant yeast strains

**Figure 1:**
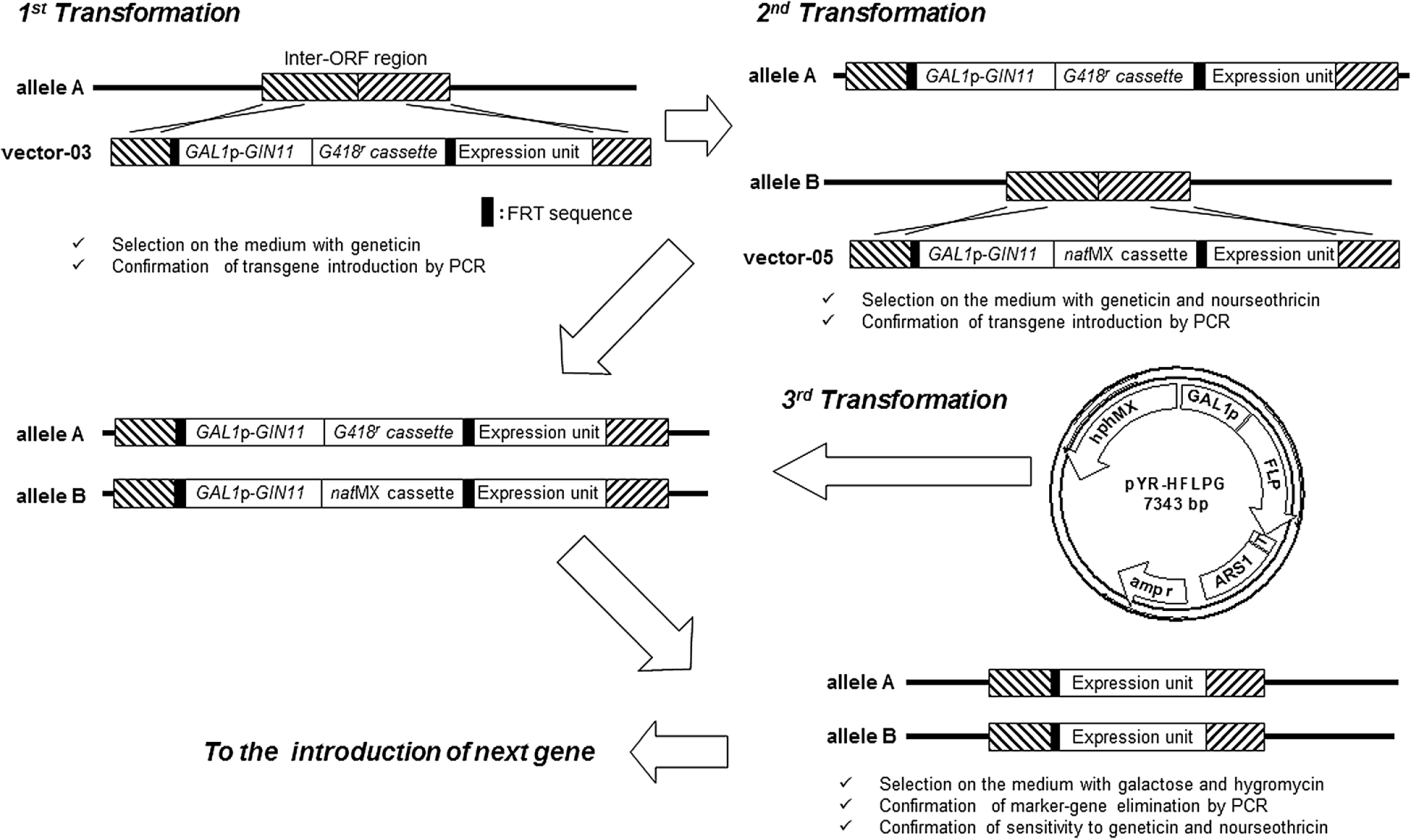
**Scheme for genetic recombination in industrial diploid yeast strains.** *GAL1*p-*GIN11*, fragment consisting of *GAL1* promoter and *GIN11*; *G418* ^*r*^ cassette, geneticin resistance gene expression unit; *nat*MX cassette, nourseothricin resistance gene expression unit; FRT, FLP recombinase recognition target; *hph*MX, hygromycin resistance gene expression unit; T, *TDH3* terminator; *amp* ^*r*^, ampicillin resistance gene expression unit.

An industrial diploid strain, sun049, was previously selected as one of superior strains for high-temperature xylose fermentation after the implementation of xylose assimilation pathway consisting of *S. stipitis* XR and XDH and overexpression of endogenous XK through genetic engineering [16], which is used for the parent strain for the multiple-gene integration in this study. The industrial sun049 does not have auxotrophic marker for the selection of transformants. Although antibiotic markers are available for the selection, the number of generally used antibiotics is limited. Consequently, a GIN11/FRT-based marker-recycling system was developed in the present study (Figure 1).

Expression units for genes of interest were integrated by homologous recombination into inter-ORF regions in *S. cerevisiae* genomic DNA with *GIN11* controlled by the *GAL1* promoter and a marker gene such as *G418*^r^ and *nat*MX, which are located between FRT sequences. To maintain gene integration and enhance the activity of enzymes encoded by the integrated genes, the expression unit was integrated in a homozygous manner with two antibiotic marker genes. Since overexpression of *GIN11* inhibits *S. cerevisiae* growth [14], induction of *GIN11* expression can promote pop-out of the markers located between the two FRT sequences. In the next step, plasmid pYR-HFLPG harboring the FLP recombinase gene and hygromycin resistance gene was transformed into the homozygous transformant. Following induction of *GIN11* and FLP recombinase gene expression by galactose, the marker genes were effectively eliminated along with *GIN11* to have sensitivity to both geneticin and nourseothiricin. Storici et al. reported that marker recycling efficiency by FLP recombination was less than 0.1% in haploid strains [15]. In the present study, using the combination of *GIN11* expression and FLP/FRT recombination, 20-100% of colonies grown on galactose-containing medium showed marker elimination. pYR-HFLPG has a chromosomally-derived autonomously replicating sequence (ARS) and is an unstable plasmid, and so can be easily cured from the genome of the recombinant *S. cerevisiae* when the yeast is grown on YPD medium without hygromycin.

The XR, XDH and XK genes were homozygously integrated to construct Sun49-1 from sun049 by the GIN11/FRT-based marker recycling system (Table 1). The ability to hydrolyze hemicellulosic materials was achieved by integrating *Trichoderma reesei XYNII* encoding endoxylanase, *Aspergillus oryzae XylA* encoding xylosidase, and *Aspergillus aculeatus BGL1* encoding β-glucosidase (BGL) into the genome of Sun49-1 to yield Sun49-5. BGL gene was integrated to hydrolyze glucooligosaccharide in the hemicellulosic fraction used in this study. Endoxylanase, xylosidase and BGL were displayed on the cell surface of the recombinant yeast strain by fusing each enzyme with the carboxy-terminal domain of α-agglutinin as an anchor region, as previously described [3]. Tolerance to fermentation inhibitors such as acetate, formate and furfural was conferred by integrating the genes for transaldolase (TAL), formate dehydrogenase (FDH), and an alcohol dehydrogenase (ADH) variant (m6ADH1 from *S. cerevisiae*)*,* into the genome of Sun49-1, yielding Sun49-24. Genes integrated into the Sun049 strain are listed in Table 1. Sun49-7 was constructed by integrating genes for hemicellulolytic enzymes and xylose-assimilating enzymes, as well as genes involved in inhibitor tolerance, into the genome of the original strain.

**Table 1:** **Characterization of** ***S. cerevisiae*** **strains used in this study**

**Strain**	**Genes integrated into the strain**
sun049	None
Sun49-1	*S. stipitis Xyl1* and *Xyl2*, *S. cerevisiae Xks1*
Sun49-5	*S. stipitis Xyl1* and *Xyl2*, *S. cerevisiae Xks1*, *T. reesei XYNII*, *A. oryzae XylA*, *A. aculeatus BGL1*
Sun49-7	*S. stipitis Xyl1* and *Xyl2*, *S. cerevisiae Xks1*, m6*ADH1*, *FDH1* and *TAL1*, *T. reesei XYNII*, *A. oryzae XylA*, *A. aculeatus BGL1*
Sun49-24	*S. stipitis Xyl1* and *Xyl2*, *S. cerevisiae Xks1*, m6*ADH1*, *FDH1* and *TAL1*

The enzymatic activities of the recombinant strains are shown in Table 2. TAL activity of Sun49-7 and Sun49-24 was 3.98-fold and 2.96-fold higher than that of Sun49-1. Sun49-7 and Sun49-24 showed 2.75-fold and 2.00-fold higher FDH activity than Sun49-1. In contrast, the TAL and FDH activities of Sun49-5 were similar that of Sun49-1. Endoxylanase, xylosidase and BGL activities were detected in Sun49-5 and Sun49-7, but not in Sun49-1 and Sun49-24 (Table 2). The activities of hemicellulolytic enzymes in Sun49-5 and Sun49-7 were similar. Functional expressions of all heterologous genes were observed in the recombinant strains, which would support the successful pop-out genome recombination.

**Table 2:** **Enzymatic activities of recombinant** ***S. cerevisiae*** **strains**

**Strain**	**Specific activity**						
	**XR [U/mg-protein]**	**XDH [U/mg-protein]**	**TAL [U/mg-protein]**	**FDH [U/mg-protein]**	**Endoxylanase [U/g-DCW]**	**Xylosidase [U/g-DCW]**	**BGL [U/g-DCW]**
Sun49-1	0.519 ± 0.033	0.979 ± 0.070	0.130 ± 0.015	0.004 ± 0.001	N.D.	N.D.	N.D.
Sun49-5	0.634 ± 0.014	1.210 ± 0.067	0.146 ± 0.022	0.004 ± 0.001	19.5 ± 2.6	94.5 ± 33.6	75.9 ± 15.8
Sun49-7	0.554 ± 0.004	1.015 ± 0.029	0.517 ± 0.018	0.011 ± 0.001	19.0 ± 3.7	86.7 ± 31.7	74.3 ± 0.7
Sun49-24	0.620 ± 0.122	1.124 ± 0.119	0.385 ± 0.010	0.008 ± 0.001	N.D.	N.D.	N.D.

Values are the averages of three independent experiments, ±SEM. N.D., not detected.

### Xylose fermentation in the presence of fermentation inhibitors

**Figure 2:**
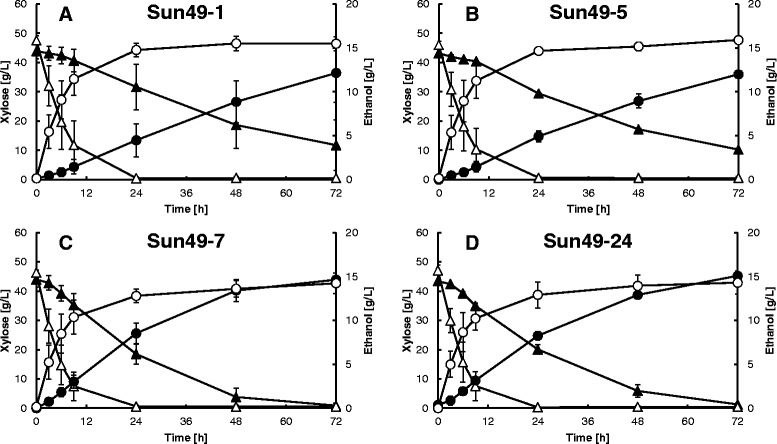
**Effect of fermentation inhibitors on xylose fermentation by** ***S. cerevisiae*** **strains Sun49-1 (A), Sun49-5 (B), Sun49-7 (C) and Sun49-24 (D).** The concentration of xylose (triangles) and ethanol (circles) was determined in the absence (open symbols) or presence (closed symbols) of 30 mM acetate, 10 mM formate and 10 mM furfural. Values are averages of three independent experiments ± SEM.

**Figure 3:**
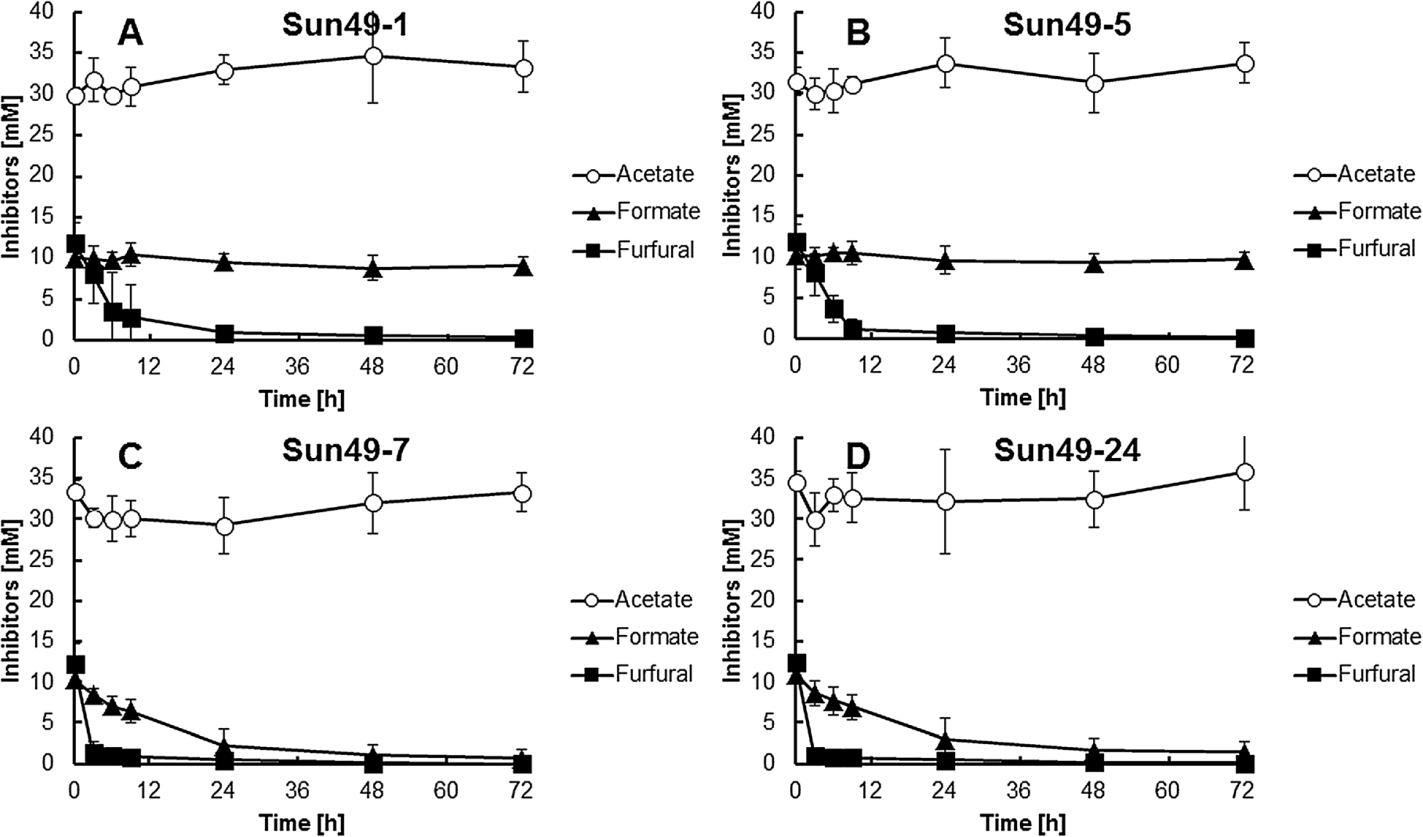
**Changes in acetate, formate and furfural concentration in the medium during xylose fermentation by** ***S. cerevisiae*** **strains Sun49-1 (A), Sun49-5 (B), Sun49-7 (C) and Sun49-24 (D).** Values are averages of three independent experiments ± SEM.

Xylose fermentation was performed in YP medium containing 50.0 g/L xylose as the sole carbon source at 30°C under oxygen limited conditions. All the recombinant strains demonstrated the same xylose fermentation ability in the absence of fermentation inhibitors (Figure 2). However, the addition of 30 mM acetate, 10 mM formate and 10 mM furfural strongly inhibited xylose consumption and ethanol production by Sun49-1 and Sun49-5. In the presence of inhibitors, after 48 h fermentation Sun49-1 and Sun49-5 consumed 25.3 g/L and 26.0 g/L xylose, respectively, (Figures 2A and D), whereas Sun49-7 completely consumed the xylose after 48 h (Figure 2C). During 24 h fermentation, Sun49-7 showed 2-fold higher volumetric ethanol productivity (0.36 g/(L.h)) compared to Sun49-1 in the presence of the inhibitors. Sun49-7 produced the same amount of ethanol in the presence or absence of the inhibitory compounds after 48 h. Figure 2D shows that Sun49-24 and Sun49-7 had similar fermentation abilities. Changes in acetate, formate and furfural concentrations during xylose fermentation are shown in Figure 3. The acetate concentration in the fermentation medium of all four recombinant strains remained essentially constant. The concentration of formate decreased in the Sun49-7 and Sun49-24 fermentation media, while Sun49-1 and Sun49-5 did not degrade formate. Furfural was metabolized by Sun49-1 and Sun49-5, because endogenous alcohol dehydrogenases can convert furfural to furfuryl alcohol [11]. Sun49-7 and Sun49-24 showed higher furfural conversion rate than Sun49-1 and Sun49-5, which was due to the functional expression of the *ADH1* variant.

### Fermentation of lignocellulosic hemicelluloses

Hydrothermal pretreatment solubilizes the hemicellulosic component of rice straw to yield liquid-soluble fraction containing mono-, oligo-, and polysaccharides [3]. Major sugars in the hemicellulosic fraction were xylose (1.26 g/L), xylobiose (0.75 g/L), glucose (0.29 g/L), cellobiose (0.08 g/L) and cellotriose (3.41 g/L), as described in the Methods section (Table 3). The amount of each constituent sugar in the hemicellulosic fraction was estimated by fully hydrolyzing the hemicelluloses with the hemicellulase reagent, Amano-90. The activities of endoxylanase, xylosidase, and BGL in Amano-90 were 650.0, 0.02, and 82.7 U/g, respectively. After 72 h hydrolysis at 50°C, the oligosaccharides and polysaccharides were degraded to 5.81 g/L xylose, 6.58 g/L xylobiose, 9.92 g/L glucose and 0.24 g/L cellobiose. Hydrolysis increased the total monosaccharides and oligosaccharides from approximately 7.20 g/L to 22.54 g/L, indicating that at least 15.34 g/L polysaccharide was contained in the hemicellulosic fraction. The hydrolysate contained fermentation inhibitors generated by the over-degradation of rice straw during hydrothermal pretreatment; the major components were acetate (28.6 mM), formate (17.6 mM) and furfural (12.6 mM) (Table 4).

**Table 3:** **Sugar content of pretreated rice straw hemicelluloses**

	**Hydrolysis treatment with hemicellulase**	
**Sugar [g/L]**	**-**	**+**
Xylose	1.26	5.81
Xylobiose	0.75	6.58
Xylotriose	0.52	ND
Xylotetraose	0.39	ND
Xylopentaose	0.30	ND
Xylohexaose	0.19	ND
Glucose	0.29	9.92
Cellobiose	0.08	0.24
Cellotriose	3.41	ND
Total	7.20	22.54

Values are averages of three independent experiments and relative standard deviations were less than 10%. N.D., not detected.

**Table 4:** **Inhibitor concentration in pretreated rice straw hemicelluloses**

**Inhibitor**	**Concentration [mM]**
Acetate	28.6
Formate	17.6
Furfural	12.6
5-HMF	0.9
Vanillin	0.3

Values are averages of three independent experiments and relative standard deviations were less than 10%.

**Figure 4:**
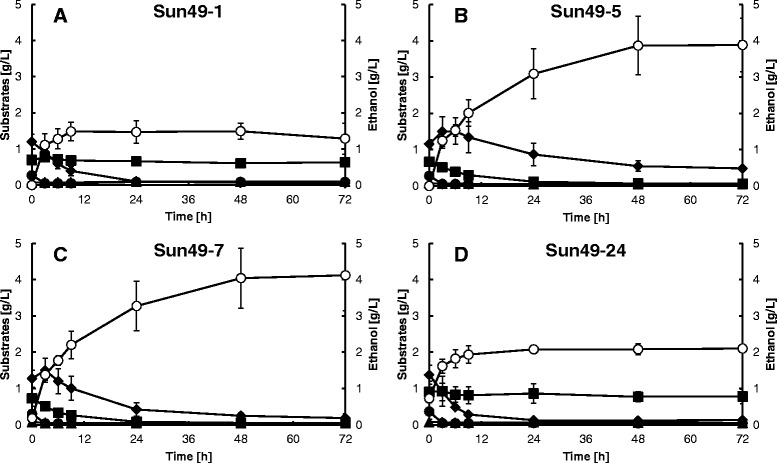
**Ethanol production from lignocellulosic hemicelluloses without addition of hemicellulase by** ***S. cerevisiae*** **strains Sun49-1 (A), Sun49-5 (B), Sun49-7 (C) and Sun49-24 (D).** Symbols: closed diamonds, xylose; closed squares, xylobiose; closed circles, glucose; closed triangles, cellobiose; open circles, ethanol. Values are averages of three independent experiments ± SEM.

The strains constructed in this study were used to ferment lignocellulosic hemicelluloses (Figure 4). Sun49-5, Sun49-7 and Sun49-24 produced 3.87 g/L, 4.04 g/L and 2.08 g/L of ethanol after 48 h fermentation, respectively, compared to 1.49 g/L for Sun49-1. Glucose was rapidly consumed by all the strains. Sun49-5 and Sun49-7 display hemicellulolytic enzymes on their cell surface; the concentration of xylose in their fermentation medium initially increased for 3 h, then decreased thereafter. (Figures 4B and C). Sun49-5 and Sun49-7 consumed xylobiose (Figures 4B and C) whereas Sun49-1 and Sun49-24 did not. Titer of ethanol produced by Sun49-7 was higher than the concentration of initial monosaccharides, indicating the functional hydrolysis of hemicelluloses by enzymes displayed on the recombinant yeast cell surface. Sugar consumption was thus improved by cell surface engineering. Sun49-7 showed higher consumption of xylose than Sun49-5, due to the expression of *TAL1*, *FDH1* and the *ADH1* variant. The yeast cell concentrations remained essentially constant throughout the fermentations, regardless of the strain (Additional file 1).

**Figure 5:**
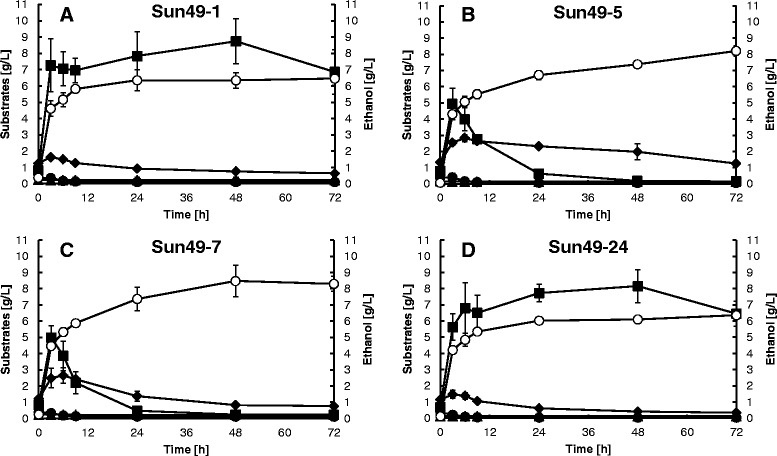
**Ethanol production from lignocellulosic hemicelluloses with addition of 0.5% (w/v) hemicellulase by** ***S. cerevisiae*** **strains Sun49-1 (A), Sun49-5 (B), Sun49-7 (C) and Sun49-24 (D).** Symbols: closed diamonds, xylose; closed squares, xylobiose; closed circles, glucose; closed triangles, cellobiose; open circles, ethanol. Values are averages of three independent experiments ± SEM.

Figure 5 shows the results of fermentation of hemicellulosic materials in the presence of 0.5% (w/v) hemicellulase reagent, Amano-90. Ethanol production was enhanced in all four strains by the addition of the commercial enzyme. The xylobiose concentration in the fermentation medium of all four strains increased after 3 h, then decreased in the Sun49-5 and Sun49-7 media due to consumption following expression of the xylosidase gene. The highest ethanol production after 48 h fermentation was achieved by Sun49-7, which expresses hemicellulolytic enzymes, xylose-assimilating enzymes, and inhibitor resistance genes: using a combination of cell surface modification and inhibitor tolerance, ethanol production was improved from 6.34 g/L to 8.48 g/L.

**Figure 6:**
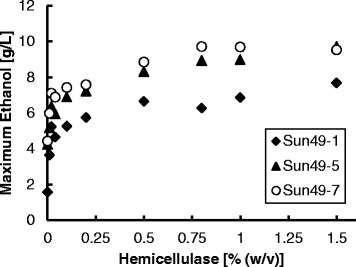
**Effect of hemicellulase concentration on ethanol production from lignocellulosic hemicelluloses by Sun49-1, Sun49-5 and Sun49-7.**

The effect of hemicellulase concentration on ethanol production is shown in Figure 6. Ethanol production showed a positive correlation with the concentration of hemicellulase in the fermentation medium, up to approximately 0.75% (w/v). Xylose released from hemicelluloses was completely consumed during the fermentation. Sun49-7 provided the highest ethanol production. Sun49-1 did not produce as much ethanol as Sun49-5 and Sun49-7, even when the concentration of hemicellulase was increased.

## Discussion

Hydrothermally pretreated hemicelluloses contain various fermentation inhibitors, including acetic acid, formic acid, and furan derivatives. Although methods for detoxifying lignocellulose hydrolysates are being developed [17,18], large-scale detoxification is technically complex and increases the cost of the fermentation process. In the present study, multiple genes encoding XR, XDH, XK, endoxylanase, xylosidase, BGL, TAL, FDH and an ADH1 variant were functionally expressed in a recombinant diploid *S. cerevisiae* strain. The multifunctional strain, developed using the GIN11/FRT-based marker recycling system, could efficiently utilize hemicelluloses to produce ethanol without any detoxification treatment.

Industrial *S. cerevisiae* strains can be engineered for lignocellulosic ethanol production because they have adapted to a wide range of environmental stresses in industrial processes [19]. Most industrial strains are diploid or polyploid, which are more tolerant to fermentation inhibitors than haploid strains [20,21]. On the other hand, modification of industrial strains requires the use of antibiotic markers due to the lack of auxotrophy. Previously, a spontaneous loss-of-heterozygosity (LOH) strategy was used to obtain transformants carrying expression cassettes homozygously [22]. However, LOH efficiency is generally low when the expression cassette further burdens yeast metabolism. In the present study, geneticin and nourseothricin resistance genes were used to select homozygous recombinants to avoid using the LOH strategy. The two antibiotic markers were simultaneously removed by galactose-induced expression of *GIN11* and the FLP recombinase gene prior to the next round of selection. FRT sequences flanking a marker gene were previously used for a 2-μm DNA-based multiple gene disruption in yeast strains [15]. In the present study, the combination of *GIN11* and FRT enabled efficient counter selection for diploid industrial strains. Moreover, an YRp-type plasmid developed in this study, pYR-HFLPG, was suitable for sequential gene integration as it is easily cured from yeast strains by removing hygromycin from the medium. This plasmid was especially useful for sun49 because it lacks 2-μm DNA. To the best of our knowledge, this is the first report demonstrating improved ethanol production from hemicellulosic polysaccharide by modifying the metabolism of a diploid yeast strain by marker recycling techniques.

As shown in Figure 3, only the recombinant strains expressing inhibitor tolerance genes quickly detoxified furfural and formate, which were likely converted to furfuryl alcohol and carbon dioxide, respectively [9,11]. Ethanol production and xylose consumption were significantly improved in xylose fermentation by the expression of *TAL1*, *FDH1* and *m6ADH1* (Figure 2). Sun49-7 showed higher xylose consumption than Sun49-5 (Figure 5) in the fermentation of lignocellulosic materials, which is due to the expression of inhibitor-tolerance genes. After 48 h fermentation, Sun49-7 produced 8.48 g/L ethanol, while ethanol produced in Sun49-5 was 7.38 g/L. This result indicates the importance of the improvement of tolerance to fermentation inhibitors for ethanol production from the hemicellulosic fraction.

Commercially-viable lignocellulosic ethanol production requires consolidation of enzyme production, saccharification, and fermentation processes to reduce costs, energy consumption, and the number of processing steps [23]. Recombinant microorganisms capable of producing ethanol directly from hemicelluloses have been developed, but few reports of hemicellulose-utilizing yeast strains have been published to date [3,24]. In the present study, a recombinant *S. cerevisiae* strain exhibiting xylose assimilation, inhibitor tolerance, and displaying hemicellulolytic enzymes on the cell surface was developed. The strain exhibiting all these attributes, Sun49-7, demonstrated the highest ethanol production from hydrothermally pretreated hemicelluloses among the strains constructed in this study (Figures 4 and 5). The addition of large amount of hemicellulase reagents into the fermentation should be avoided for the cost-effective ethanol production. This study demonstrated that the improvement of yeast tolerance to fermentation inhibitors and display of hemicellulase on the yeast cell surface could reduce the addition of commercial enzymes as shown in Figure 6. Further enhancement in the activity of hemicellulolytic enzymes displayed on yeast cell surface would contribute to further reduction of ethanol cost.

Previously, close proximity of multiple cellulases on the cell surface enabled synergistic hydrolysis of cellulose, which led to increased sugar availability for ethanol production [25,26]. Figure 6 shows that the xylose-utilizing strain did not produce as much ethanol as the hemicellulolytic strain, even at high hemicellulase concentration, indicating that enzymes displayed on the yeast cell surface can hydrolyze hemicelluloses that are not hydrolyzed by commercial hemicellulases. Xylose released from hemicelluloses was completely consumed during the fermentation (Figures 4 and 5). These data demonstrate the advantage of cell surface engineering for increased hemicellulose hydrolysis for improved ethanol production.

## Conclusions

The combination of *GIN11* expression and FLP/FRT recombination enabled efficient counter selection for multiple gene integration into the genome of an industrial diploid *S. cerevisiae* strain. Homozygous integration of nine genes conferring hemicellulose-hydrolysis and xylose-assimilation capabilities, and inhibitor tolerance, enabled direct conversion of the hemicellulosic material obtained by the hydrothermal pretreatment of rice straw to ethanol. The multiple gene integration technique developed here would be applicable for the synthesis of metabolic pathways in recombinant yeast strains through a synthetic biology approach as well as the development of biomass utilizing yeast strain for consolidated bioprocessing.

## Methods

### Microbial strains and media

Yeast strains were routinely cultivated at 30°C in synthetic medium [SD medium; 6.7 g/L of yeast nitrogen base without amino acids (Difco Laboratories, Detroit, MI), 20 g/L of glucose] supplemented with antibiotics, and in YPD medium (20 g/L peptone, 10 g/L yeast extract, 20 g/L glucose). *Escherichia coli* NovaBlue (Novagen, Inc., Madison, WI) was used as the host strain for recombinant DNA manipulation. *E. coli* was grown in Luria-Bertani medium (10 g/L peptone, 5 g/L yeast extract, and 5 g/L sodium chloride) containing 100 mg/L ampicillin.

### Plasmid construction

Multiple cloning site (MCS) linker 1, made with oligonucleotides P1 and P2, was inserted into the *Pvu*II-*Pci*I site of pBluescriptSK + ® (Agilent Technologies, Palo Alto, CA). All oligonucleotides used for plasmid construction and PCR are shown in Additional file 2. Schematic representation of plasmid construction is shown in Additional file 3. *GIN11* and *GAL1* promoter were obtained by PCR with primer sets P3/P4 and P5/P6, respectively, using genomic DNA from *S. cerevisiae* X2180-1A [27] as a template. The *GIN11* and *GAL1* promoter were inserted into the *Spe*I-*Eco*RV site of MCS linker 1. The geneticin resistance gene (*G418*^r^) and nourseothricin resistance gene (*nat*MX) were obtained from pYC030 and pYC050 [28] by digestion with *Asc*I and inserted into the *Asc*I site adjacent to *GIN11*, yielding pBGIN11-03 and pBGIN11-05, respectively. *GIN11* is on chromosome XIII 923146 to 923827, and identified as a gene that leads *S. cerevisiae* cells to die when overexpressed [14]. P4 and P5 contain FRT sequences encoded on a 2-μm plasmid [15]. *GAL1* promoter, *GIN11,* and a marker gene were located between the FRT sequences in pBGIN11-03 and pBGIN11-05. The expression units X1X2XKN2, XYNII, BGL1XYLA, and m6ADH1FDH1TAL1 were inserted into the *Mlu*I and *Not*I site of pBGIN11-03 and pBGIN11-05, yielding expression unit vector-03 and vector-05, respectively. Each expression unit was derived as follows. *PYK1* terminator was obtained by PCR using primer set P7/P8, then was replaced with *TDH3* terminator adjacent to the *XYL2* of pIUX1X2XKN [16], yielding pIUX1X2XKN2. The expression unit X1X2XKN2 was obtained from pIUX1X2XKN2 by *Bss*HII digestion. The expression unit XYNII was obtained from pdU-GPAGXynII [3] by *Not*I digestion. The expression unit BGL1XYLA was obtained from pIHPGBGGPXylA [3] by *Bss*HII digestion. Three expression cassettes, m6*ADH1*, *FDH1*, and *TAL1,* were obtained by *Not*I and *Psp*OMI digestion from pEWm6ADH1 [11], pGK423-*FDH1* [9], and pGK404ScTAL1 [8], respectively. These three cassettes were inserted into *Not*I of pBGIN11-03 and pBGIN11-05 in tandem.

MCS linker 2, made with oligonucleotides P9 and P10, was inserted into the *Pvu*II site of pBluescriptSK + ®. Four inter-ORF regions on genomic DNA, I2, I6, I7S, and I10, were obtained by PCR using X2180-1A as template DNA and the primer sets P11/P12, P13/P14, P15/P16, and P17/P18, respectively, and inserted into the *Not*I site of MCS linker 2. The I2, I6, I7S, and I10 regions are located between YFL021W and YFL020C, YGR249W and YGR250C, YKL219W and YKL218C, and YPL257W and YPL256C, respectively. The inter-ORF regions are situated downstream of both adjacent ORFs. Linker 1 consisting of P19 and P20 was introduced into the *Xba* I site of I2 region, the blunt ended *Bam*HI-*Eco*RV site of I6 region, and the *Bsa*AI site of I7S region. Linker 2 consisting of P21 and P22 was inserted into the *Xba* I site of the I10 region. The four resulting pIX + Linker plasmids were digested by *Apa*I and ligated with expression unit vector-03 (or −05) digested by *Apa*I or *Pci*I to generate the integration plasmids. The vectors prepared for integration into the genome of *S. cerevisiae* were referred to as pI2X1X2XKN2-03, pI2X1X2XKN2-05, pI6XYNII-03, pI6XYNII-05, pI7SBGL1XYLA-03, pI7SBGL1XYLA-05, pI10m6ADH1FDH1TAL1-03, and pI10m6ADH1FDH1TAL1-05.

To make pYR-HFLPG, a partial fragment of pBR322 [29] containing *amp*^*r*^ and ori was obtained by PCR using the primer set P23/P24, which was subsequently self-ligated. Hygromycin resistant gene (*hph*MX) was obtained by *Asc*I digestion of pYC040 [28] and ARS1 was obtained by *Eco*RI-*Hind*III digestion of YRp7 [30]; *hph*MX and ARS1 were inserted into the *Asc*I site and *Eco*RI-*Hin*dIII site, respectively, of the self-ligated fragment. The resultant plasmid was digested with *Spe*I and *Hin*dIII. *GAL1* promoter (*Spe*I-*Bam*HI), *FLP* gene (*Bam*HI-*Sal*I) and *TDH3* terminator (*Sal*I-*Hin*dIII) were introduced in tandem, yielding pYR-HFLPG. *GAL1* promoter and *FLP* gene were obtained by PCR using X2180-1A as template DNA and primer sets P25/P26 and P27/P28, respectively. *TDH3* terminator was obtained from pUP3GLP [31] by *Sal*I-*Hin*dIII digestion.

### Yeast transformation

The industrial *S. cerevisiae* strain Sun049 was obtained from Suntory Limited (Tokyo, Japan) [16]. The vector pI2X1X2XKN2-03 was digested by *Fse*I. Both ends of the resultant linear fragment contained an inter-ORF region I2; this fragment was introduced into sun049 as the first transformation. The first transformants were selected on YPD containing 300 μg/mL geneticin. The integration of the expression unit into the I2 region of the *S. cerevisiae* genome was confirmed by PCR using the primers P29, P30, P32 and P33. P29 and P32 are the sequences located upstream and downstream of I2 region, respectively, and P30 and P33 are the sequences within the transformed linear fragment, *G418*^r^ and X1X2XKN, respectively. The second transformation, with pI2X1X2XKN2-05, was performed using the first transformant as the host. Selection was conducted on YPD medium containing 300 μg/mL geneticin and 50 μg/mL nourseothricin. Homozygous integration into the I2 region was confirmed by PCR using a primer set, P29/P31. pYR-HFLPG was transformed into the second transformant to eliminate the antibiotic marker genes in the resulting third transformant. The third transformants were selected on YP medium (20 g/L peptone, 10 g/L yeast extract) containing 20 g/L galactose and 200 μg/mL hygromycin. As the expression of *GIN11* leads cells to die, only cells in which homologous recombination between the two FRT sequences occurred should be able to grow on medium containing galactose. The expression of FLP recombinase promotes homologous recombination. Introduction of pYR-HFLPG should confer marker recycling in strain sun049, which lacks a 2-μm plasmid carrying FLP recombinase. Marker elimination was verified by colonies being sensitive to geneticin and nourseothricin, and by PCR using the primer set P34/P35. pYR-HFLPG has a chromosomally-derived ARS and is an unstable plasmid; consequently, it can be easily cured from the host when the yeast is grown on YPD without hygromycin. This procedure provided Sun49-1. Similarly, Sun49-5 was constructed from Sun49-1 using plasmids pI6XYNII-03/-05 and pI7SBGL1XYLA-03/-05, Sun49-7 was constructed from Sun49-5 by transformation with pI10m6ADH1FDH1TAL1-03/-05, and Sun49-24 was constructed from Sun49-1 by transformation with pI10m6ADH1FDH1TAL1-03/-05. P36 to P50 were used in order to verify the integration of the expression unit or elimination of the marker gene during the construction of Sun49-5, Sun49-7 and Sun49-24.

### Enzyme assays

After cultivation in YPD medium for 48 h at 30°C, cells were harvested by centrifugation at 6000 × *g* for 5 min at 4°C and washed with 10 mM potassium phosphate buffer (pH7.5) containing 2 mM EDTA. Then, the cells were resuspended in 100 mM potassium phosphate buffer (pH7.5) containing 2 mM MgCl_2_ and 2 mM dithiothreitol. The suspended cells were mixed with glass beads (0.5 mm diameter), disrupted by shaking at 2500 rpm at 4°C for 5 min with a Multi-beads shocker (Yasui Kikai Corporation, Osaka Japan). The cell extract, collected after centrifugation at 15000 × *g* for 5 min at 4°C, was used for the enzyme assay. XR activity was measured spectrophotometrically by monitoring NADPH oxidation at 340 nm in a reaction mixture with the following composition: 100 mM sodium phosphate buffer (pH7.0) at 30°C, 200 mM xylose, and 0.24 mM NADPH. XDH activity was measured spectrophotometrically by monitoring NAD^+^ reduction at 340 nm in a reaction mixture with the following composition: 100 mM Tris–HCl (pH7.0) at 30°C, 1 mM MgCl_2_, 50 mM xylitol, and 2 mM NAD^+^. The activities of TAL and FDH were measured as previously described [8,9]. One unit of enzyme activity was defined as the amount of enzyme that oxidized or reduced 1 μmol of NADPH or NAD^+^ per minute. The activities of endoxylanase, xylosidase and BGL on the yeast cell surface were determined as described previously [3]. One unit of enzyme activity was defined as the amount of enzyme that released 1 μmol of the product from the substrate per minute. Protein concentrations were determined using a protein assay kit (Bio-Rad, Hercules, CA) using bovine serum albumin as the standard.

### Xylose fermentation under oxygen-limited conditions

Following cultivation under aerobic conditions for 48 h at 30°C in YPD medium, yeast cells were collected by centrifugation at 1000 × *g* for 5 min at 4°C, washed twice with distilled water, and then inoculated into YPX medium (10 g/L yeast extract, 20 g/L peptone, and 50 g/L xylose) with or without addition of an inhibitor mixture consisting of 30 mM acetate, 10 mM formate and 10 mM furfural. The initial cell concentration was adjusted to 50 g wet cells/L. Wet cell weight was determined by weighing a cell pellet that was harvested by centrifugation at 1,000 × g for 5 min. The estimated dry cell weight (DCW) was approximately 0.15-fold the wet cell weight. All fermentations were performed at 30°C under oxygen limited conditions at an agitation speed of 500 rpm in 100-mL closed bottles equipped with a bubbling CO_2_ outlet and a stir bar, as described previously [32]. Cell growth was monitored by absorbance at 600 nm. Wet cell weight was determined by weighing a cell pellet harvested by centrifugation at 1000 × *g* for 5 min. The concentrations of acetate, ethanol, formate, glycerol, xylitol, and xylose in the fermentation medium were determined as described previously [10]. Furfural concentrations in the fermentation media were determined according to a previous method [11].

### Fermentation of lignocellulosic hydrolysate

A hemicellulosic fraction obtained by liquid hot water treatment of rice straw at 130-300°C under the pressure of less than 10 MPa was purchased from Mitsubishi Heavy Industries, Ltd. (Tokyo Japan). The hemicellulosic fraction was separated from solid cellulose-enriched fraction by filtration and adjusted to pH 5 using NaOH as described previously [10], which contained 0.29 g/L glucose, 0.08 g/L cellobiose, 3.41 g/L cellotriose, 1.26 g/L xylose, 0.75 g/L xylobiose, 0.52 g/L xylotriose, 0.39 g/L xylotetraose, 0.30 g/L xylopentaose, 0.19 g/L xylohexaose as mono- and oligo-saccharides, and 28.6 mM acetate, 17.6 mM formate, 12.6 mM furfural, 0.9 mM 5-HMF and 0.3 mM vanillin as fermentation inhibitors. The sugar components of the hemicellulosic material were analyzed by hydrolyzing the polysaccharides and oligosaccharides with 10 g/L commercial hemicellulase (Amano-90; Amano Enzyme, Nagoya, Japan) by shaking at 25 rpm at 50°C for 72 h. The activities of endoxylanase, xylosidase, and BGL in the hemicellulase were 650.0, 0.02, and 82.7 U/g, respectively. Proteins were removed from the hydrolysate by filtration through an Amicon Ultra filter (MWCO 10 kDa, Millipore, MA), then the sugar content was measured using a high performance liquid chromatography-evaporative light scattering detector [3]. The hemicellulosic fraction was supplemented with 10 g/L yeast extract and 20 g/L peptone, 50 wet-g/L yeast cells prepared as described above were added, then fermentation was conducted for 72 hours. The concentrations of mono- and oligo-saccharides in the fermentation medium were measured by gas chromatography–mass spectrometry after derivatization, as described previously [3]. The concentration of ethanol was quantified as described above.

## Additional files

Additional file 1:
**Cell concentration during the fermentation of lignocellulosic hemicelluloses by Sun49-1, Sun49-5, Sun49-7 and Sun49-24.** Values are the averages of three independent experiments. Additional file 2:
**Oligonucleotides used in this study.** FRT sequences are underlined. Additional file 3:
**Schematic representation of plasmid construction.** Expression units: X1X2XKN2 containing *S. stipitis Xyl1* and *Xyl2*, and *S. cerevisiae Xks1*; XYNII containing *T. reesei XYNII*; BGL1XYLA containing *A. aculeatus BGL1* and *A. oryzae XylA*; m6ADH1FDH1TAL1 containing *S. cerevisiae ADH1* variant, *FDH1* and *TAL1*.

## Abbreviations

ADH: Alcohol dehydrogenase
ARS: Autonomously replicating sequence
BGL: β-glucosidase
FDH: Formate dehydrogenase
FRT: Flp recombinase recognition target
GIN: Growth-inhibitory sequence
5-HMF: 5-hydroxymethylfurfural
MCS: Multiple cloning site
TAL: Transaldolase
XDH: Xylitol dehydrogenase
XK: Xylulokinase
XR: Xylose reductase
