# Supplementary figures and images for: Development of a GIN11/FRT-based multiple-gene integration technique affording inhibitor-tolerant, hemicellulolytic, xylose-utilizing abilities to industrial Saccharomyces cerevisiae strains for ethanol production from undetoxified lignocellulosic hemicelluloses
Source: Microb Cell Fact. 2014 Oct 12;13:145. doi: 10.1186/s12934-014-0145-9 (PMC4198627; doi:10.1186/s12934-014-0145-9)

# Additional file 1

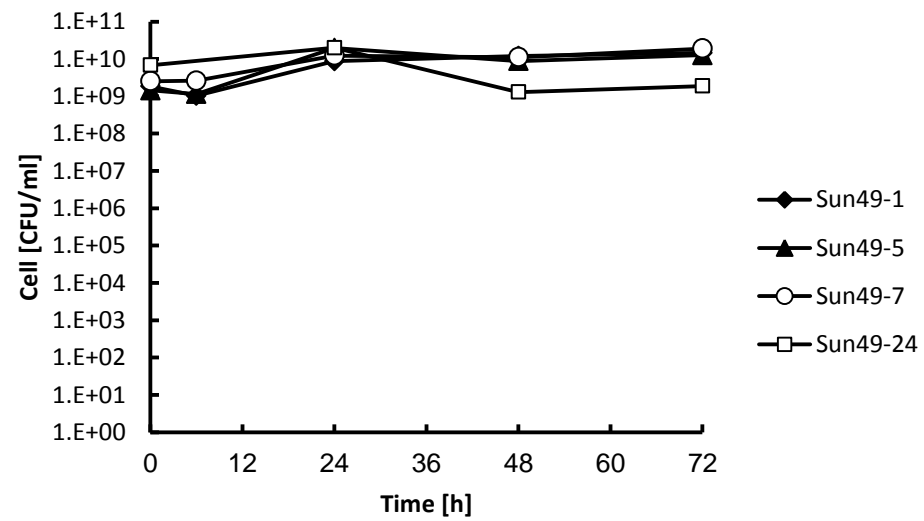

Supplement: Additional file 1: — Cell concentration during the fermentation of lignocellulosic hemicelluloses by Sun49-1, Sun49-5, Sun49-7 and Sun49-24. Values are the averages of three independent experiments. [file 12934_2014_145_MOESM1_ESM.pdf]
