# Supplementary material for: Development of a GIN11/FRT-based multiple-gene integration technique affording inhibitor-tolerant, hemicellulolytic, xylose-utilizing abilities to industrial Saccharomyces cerevisiae strains for ethanol production from undetoxified lignocellulosic hemicelluloses
Source: Microb Cell Fact. 2014 Oct 12;13:145. doi: 10.1186/s12934-014-0145-9 (PMC4198627; doi:10.1186/s12934-014-0145-9)
Supplement: Additional file 2: — Oligonucleotides used in this study. FRT sequences are underlined. [file 12934_2014_145_MOESM2_ESM.docx]

**Additional file 2** Oligonucleotides used in this study

| Oligonucleotide | Sequence |
| --- | --- |
| P1 | 5’-ACATGTGGGCCCACTAGTGATATCACGCGTAGCGCTGCGGCCGCGGGCCCA-3’ |
| P2 | 5’-CATGTGGGCCCGCGGCCGCAGCGCTACGCGTGATATCACTAGTGGGCCCACATGT-3’ |
| P3 | 5’-GGATCCGGAATTTCGATCAATAACAGTGTTTGTG-3’ |
| P4 | 5’GATATCTGCAGAATTCGCCCTTGAAGTTCCTATACTTTCTAGAGAATAGGAACTTCGGCGCGCCGTCGACCTGCAGACTAGATGCACTCATAT-3’ |
| P5 | 5’-ACTAGTGAATTCTCCTATTCTCTAGAAAGTATAGGAACTTCGTCAATTCGACAGGTTATCAGCA-3’ |
| P6 | 5’-GGATCCTATAGTTTTTTCTCCTTGACG-3’ |
| P7 | 5’-GGATCCAAAAAGAATCATGATTGAATGAAGATAT-3’ |
| P8 | 5’-CGAGATTGACATAGAGTCAA TTTAAATACTAGT-3’ |
| P9 | 5’TTAATTAACAGCTGGGCCGGCCGTTTAAACGCGGCCGCGTTTAAACGGCCGGCCCAGCTGGCGCCTTAATTAAGCCCGGGCATTTAAAT-3’ |
| P10 | 5’ATTTAAATGCCCGGGCTTAATTAAGGCGCCAGCTGGGCCGGCCGTTTAAACGCGGCCGCGTTTAAACGGCCGGCCCAGCTGTTAATTAA-3’ |
| P11 | 5’-CGGCCGTTTAAACGCAGTACTCGCTTCTTTCCATG-3’ |
| P12 | 5’-CGTTTAAACGCGGCCGCGATAGATTCCCTTAGGGA-3’ |
| P13 | 5’-CGGCCGTTTAAACGCATTATTTTCAACAGAACACA-3’ |
| P14 | 5’-CGTTTAAACGCGGCCATTGCATCAGGTCCATAAAAT-3’ |
| P15 | 5’-CGGCCGTTTAAACGCCTCTGGTTAACAAAAACCAG-3’ |
| P16 | 5’-CGTTTAAACGCGGCCGATTTTGAAGTGAGTTCAGC-3’ |
| P17 | 5’-CGGCCGTTTAAACGCTGGAATGGTAGTCGAAAGGT-3’ |
| P18 | 5’-CGTTTAAACGCGGCCTTCCTGTTCTTTTCATGATA-3’ |
| P19 | 5’-CCATGGGCCC-3’ |
| P20 | 5’-GGGCCCATGG-3’ |
| P21 | 5’-CTAGCCATGGGCCC-3’ |
| P22 | 5’-CTAGGGGCCCATGG-3’ |
| P23 | 5’-GTTTAAACGGATCCACTAGTAAGCTTGAATTCTTGAAGACGAAAGGG-3’ |
| P24 | 5’-GGCGCGCCGTTTAAACGGTACCCCTGCCTCGCGCGTTTCGGTG-3’ |
| P25 | 5’-GGCGCGCCGTTTAAACGGATCCACTAGTGAATTCTCCTATTC-3’ |
| P26 | 5’-GGATCCTATAGTTTTTTCTC-3’ |
| P27 | 5’-GGATCCCTCGAGGCGGCCGCATGCCACAATTTGGTATATTATG-3’ |
| P28 | 5’-GTCGACTCTAGAGAATAGGAACTTCGGAATAGGAACTTCA-3’ |
| P29 | 5’-AACACCACTCCAAATGGC-3’ |
| P30 | 5’-CGTTTCCCGTTGAATATGGC-3’ |
| P31 | 5’-GTGTCGTCAAGAGTGGTACC-3’ |
| P32 | 5’-GGCTGAATACTACTCCTTCCA-3’ |
| P33 | 5’-TGGGGCTTCTAAAAACGATG-3’ |
| P34 | 5’-AACACCACTCCAAATGGC-3’ |
| P35 | 5’-TTCACGCTTGACGATACC-3’ |
| P36 | 5’-TATTAACAGCGCATCGCAAG-3’ |
| P37 | 5’-AGCCAATTGTCCGGTATCC-3’ |
| P38 | 5’-CAATGTTCCCATCATTTGGG-3’ |
| P39 | 5’-TATTAACAGCGCATCGCAAG-3’ |
| P40 | 5’-AAAGTTGCCCGAGTTGGAC-3’ |
| P41 | 5’-CCCGTCTACTGTTAATCAAGG-3’ |
| P42 | 5’-AGCCAATTGTCCGGTATCC-3’ |
| P43 | 5’-CTTGTTTGGGATTTGCAGG-3’ |
| P44 | 5’-CCCGTCTACTGTTAATCAAGG-3’ |
| P45 | 5’-GTTCCGGTGGTCAGGTTG-3’ |
| P46 | 5’-GAGATTCGCGACGAAGGTT-3’ |
| P47 | 5’-CCGGCGACAAGATTTCTTAC-3’ |
| P48 | 5’-CCACCCAAGACAAGCTTCA-3’ |
| P49 | 5’-GAGATTCGCGACGAAGGTT-3’ |
| P50 | 5’-CCCGATGTATGGGTTTGG-3’ |

FRT sequences are underlined.
