# Supplementary material for: Development of a GIN11/FRT-based multiple-gene integration technique affording inhibitor-tolerant, hemicellulolytic, xylose-utilizing abilities to industrial Saccharomyces cerevisiae strains for ethanol production from undetoxified lignocellulosic hemicelluloses
Source: Microb Cell Fact. 2014 Oct 12;13:145. doi: 10.1186/s12934-014-0145-9 (PMC4198627; doi:10.1186/s12934-014-0145-9)
Supplement: Additional file 3: — Schematic representation of plasmid construction. Expression units: X1X2XKN2 containing S. stipitis Xyl1 and Xyl2, and S. cerevisiae Xks1; XYNII containing T. reesei XYNII; BGL1XYLA containing A. aculeatus BGL1 and A. oryzae XylA; m6ADH1FDH1TAL1 containing S. cerevisiae ADH1 variant, FDH1 and TAL1. [file 12934_2014_145_MOESM3_ESM.pptx]

## Slide 1
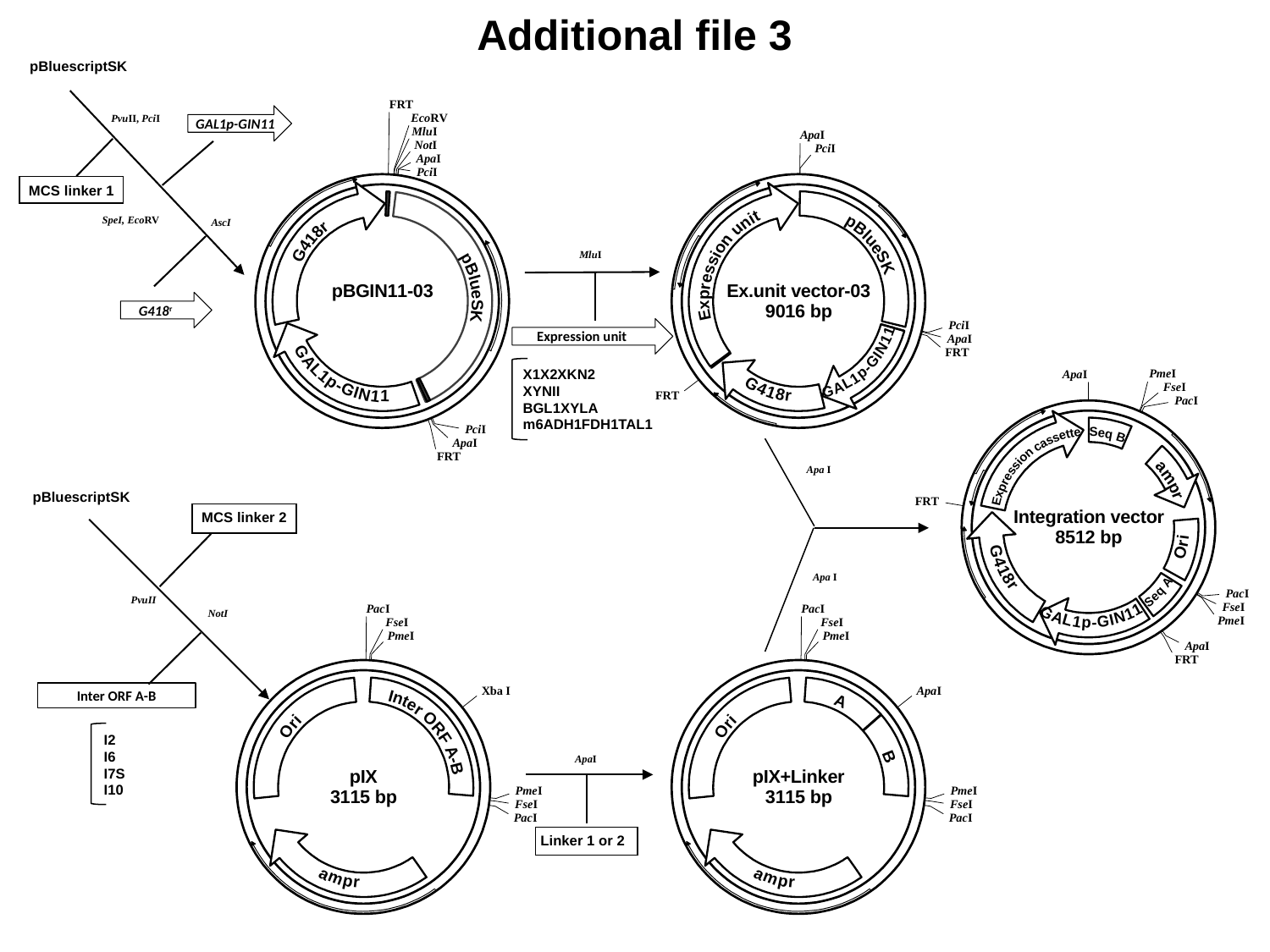

Additional file 3
pBluescriptSK
PvuII, PciI
GAL1p-GIN11
MCS linker 1
SpeI, EcoRV
AscI
MluI
G418r
Expression unit
X1X2XKN2
XYNII
BGL1XYLA
m6ADH1FDH1TAL1
Apa I
pBluescriptSK
MCS linker 2
Apa I
PvuII
NotI
Inter ORF A-B
I2
I6
I7S
I10
ApaI
Linker 1 or 2
